# Enhancing Photothermal Therapy Against Breast Cancer Cells by Modulating the End Point of Gold Shell-Isolated Nanoparticles Using Nanostraw-Assisted Injection

**DOI:** 10.1021/acsami.5c00084

**Published:** 2025-04-29

**Authors:** Sabrina A. Camacho, Pedro H. B. Aoki, Frida Ekstrand, Osvaldo N. Oliveira, Christelle N. Prinz

**Affiliations:** † School of Sciences, Humanities and Languages, São Paulo State University (UNESP), Assis, SP 19806-900, Brazil; ‡ Division of Solid-State Physics and NanoLund, Lund University, 221 00 Lund, Sweden; § São Carlos Institute of Physics, University of São Paulo (USP), São Carlos, SP 13566-590, Brazil

**Keywords:** gold shell-isolated nanoparticles, breast cancer cells, photothermal therapy, nanostraw-assisted injection, incubation

## Abstract

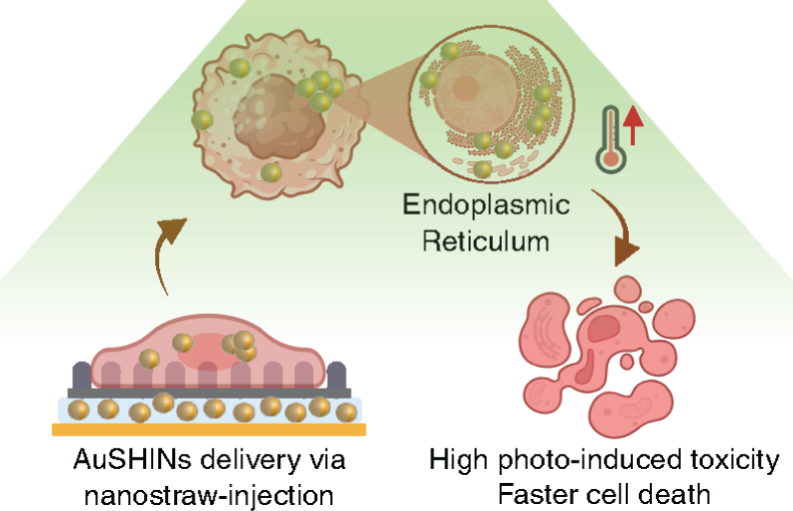

Gold shell-isolated nanoparticles (AuSHINs) are promising photothermal therapy (PTT) agents for cancer treatment due to their excellent photoconversion efficiency, biocompatibility, colloidal stability, and tunable properties, including size, shape, and surface functionalization. However, their therapeutic efficacy in *in vitro* assays is often limited by poor cellular uptake and lysosomal entrapment, which can result in nanoparticle degradation and a reduction in PTT effectiveness. In this study, we demonstrate that nanostraw-assisted injection enhances the PTT efficacy of AuSHINs compared to the conventional incubation method, as evaluated in human breast cancer cell lines: adenocarcinoma cells (MDA-MB-231) and glandular carcinoma cells (MCF7). This enhancement is attributed to three differences between the delivery methods: nanoparticle internalization, intracellular targeting, and the progression of cell death pathways. Nanostraw injection resulted in approximately 10-fold higher internalization of AuSHINs compared to 0.5-h incubation. Confocal fluorescence microscopy revealed that AuSHINs delivered via conventional incubation predominantly localize within lysosomes, whereas those introduced through nanostraw-assisted injection primarily targeted the endoplasmic reticulum (ER), thus avoiding lysosomal degradation. This differential targeting led to approximately a 2-fold higher reduction in the viability of photoactivated breast cancer cells treated with nanostraw-delivered AuSHINs. Furthermore, nanostraw-assisted injection accelerated the initiation of apoptosis relative to incubation. PTT-induced cell death was more pronounced in MCF7 cells compared to MDA-MB-231 cells, reflecting the higher resistance to therapy of the latter. These findings highlight the potential of nanostraw-assisted injection to enhance PTT, and we now face the challenge of integrating it into *in vivo* delivery strategies.

## Introduction

Light-based therapies offer a noninvasive approach to destroying malignant cells, making them increasingly attractive in clinical settings.^1−3^ In particular, photothermal therapy (PTT) is able to target and destroy cancer cells selectively through administering photothermal agents to increase the local temperature by several degrees Celsius upon light exposure, thus inducing hyperthermia in tumor cells.^3−6^ The unique characteristics of the tumor microenvironment, such as hypoxia, acidity, and nutrient deficiency, render cancer cells more susceptible to local temperature increases, thereby allowing for targeted destruction.^6−8^ Promising candidates for PTT include gold shell-isolated nanoparticles (AuSHINs) owing to their high photoconversion efficiency, excellent biocompatibility, colloidal stability, and customizable size, shape, and functionalization.^9−14^

The possible mechanisms of AuSHINs’ action have been investigated using membrane models, i.e., Langmuir monolayers,^12,13,15,16^ with the assumption that plasma membranes are important targets in PTT. However, cell death is not necessarily initiated by phenomena at the membrane; other organelles may be involved. Indeed, the intracellular fate of nanoparticles is essential since it can impact efficacy and side effects.^17^ For instance, lysosomal entrapment can lead to nanoparticle degradation, resulting in a reduced PTT effect. Therefore, determining the intracellular end point of the nanoparticles is important to design effective nanoparticle-light-based therapies.^18−20^ Another issue is the cellular internalization efficiency of AuSHINs, which may be limited when incubated with cells.^21,22^ This suggests that incubation with nanomaterials is not the most efficient method of delivery for cellular uptake. One way to deliver nanomaterials to the cytosol of multiple cells efficiently is nanostraw-assisted injection,^23,24^ a gentle method that can provide high transfection efficiency while maintaining high cell viability.^25,26^ Using this method, cells are seeded on top of a nanostraw substrate while the nanomaterial is in solution underneath the substrate. Mild electrical pulses are applied across the substrate, resulting in a local opening of pores in the cell membrane on top of the nanostraws. This method is less harmful than conventional electroporation because the electric field is focused on a much smaller area of the cell. During the application of the electrical pulses, the nanomaterials are transported through the nanostraws to the cytosol via electrophoresis.^27^ With this method, nanodiamonds have been delivered in larger quantities compared to incubation, and they were mostly located in the cytosol, thereby avoiding lysosomal entrapment.^24^

In this study, we demonstrate that the PTT efficiency of AuSHINs can be enhanced using nanostraw-assisted injection as a delivery method. Spherical AuSHINs were chosen for PTT treatment due to their lower toxicity and higher colloidal stability.^28−30^ For instance, spherical gold nanoparticles are generally more biocompatible than gold nanorods, which often require cetyltrimethylammonium bromide during synthesis—a surfactant known for its cytotoxicity.^31,32^ Human breast cancer cell lines, adenocarcinoma cells (MDA-MB-231), and glandular carcinoma cells (MCF7), were selected as therapeutic models due to the high incidence and mortality of these breast cancers, as well as their distinct characteristics. MDA-MB-231 cells are metastatic and triple-negative (ER-, PR-, and HER2-), while MCF7 cells are non-metastatic and responsive to estrogen (ER+) and progesterone (PR+).^33^ The PTT effects on cancer cells *in vitro*, induced by the photoactivation of AuSHINs delivered via incubation or nanostraw-assisted injection, were evaluated through analysis of AuSHINs internalization, cell viability, cell death pathways, reactive oxygen species (ROS) generation, and AuSHINs localization.

## Results and Discussion

### AuSHINs and AuSHINs–ATTO 647N Characterization

**Figure 1:**
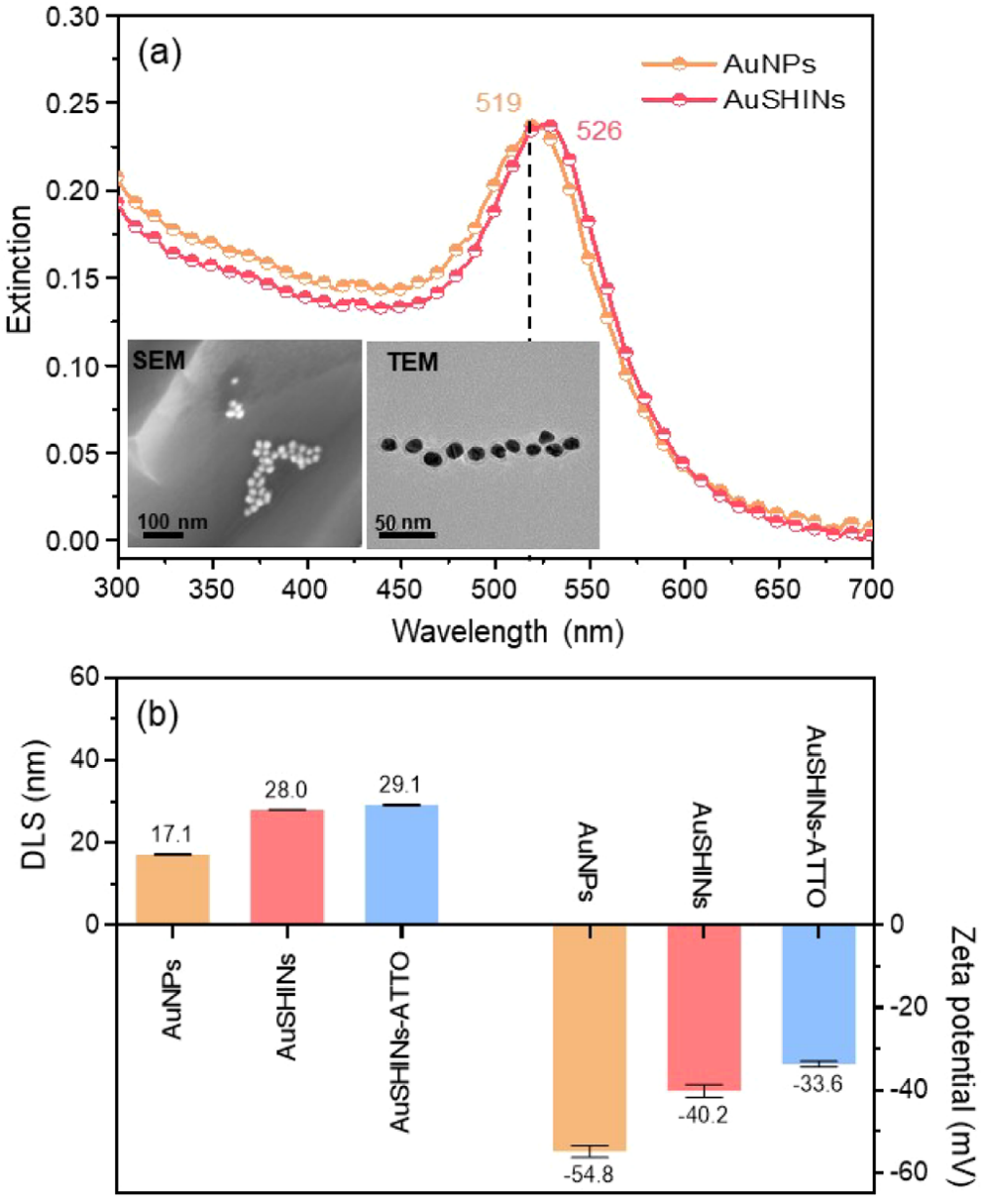
(a) UV-Vis extinction spectra of AuNPs and AuSHINs diluted in ultrapure water showing the maximum LSPR peaks at 519 and 526 nm, respectively. SEM and TEM images of AuSHINs are shown in the insets. (b) DLS average hydrodynamic diameter and ζ-potential for AuNPs, AuSHINs and AuSHINs–ATTO 647N (AuSHINs−ATTO) in ultrapure water (±standard deviation). The polydispersity index (PdI) is 0.1 for all the nanoparticles.

The ultraviolet–visible spectrophotometry (UV-Vis) extinction spectra of AuNPs and AuSHINs diluted in ultrapure water are shown in Figure 1a. The localized surface plasmon resonance (LSPR) at 519 nm for AuNPs is shifted to 526 nm for AuSHINs owing to the refractive index change caused by the silica coating.^12,14,16,34,35^ The scanning electron microscopy (SEM) and transmission electron microscopy (TEM) images in the inset confirmed the nanoparticle’s spherical shape and the presence of the silica coating. The gold core has an average diameter of 14.6 ± 1.4 nm, and the silica shell has an average thickness of 5.7 ± 1.0 nm, as determined from SEM and TEM image analysis. The average hydrodynamic diameters, evaluated using dynamic light scattering (DLS), and the ζ-potential of AuNPs, AuSHINs, and AuSHINs–ATTO 647N are displayed in Figure 1b. The AuNPs have an average hydrodynamic diameter of 17.1 ± 1.0 nm, which increases to 28.0 ± 0.3 nm with the silica coating and further to 29.1 ± 0.2 nm with the conjugation of ATTO 647N molecules. The ζ-potential was also affected by the presence of the shell and fluorophore, especially noticeable when comparing AuNPs (−54.8 ± 2.1 mV) and AuSHINs–ATTO 647N (−33.6 ± 0.5 mV). The increase in the ζ-potential is related to the reduction of negative charges around the nanoparticles. Indeed, AuNPs are negatively charged^36^ and the inert silica shell^16,37^ and positive ATTO 647N molecules decrease the surface charge.

The colloidal stability of AuSHINs was verified in ultrapure water, 10x diluted PBS, and complete DMEM and RPMI cell culture media (containing 10% FBS and 1% penicillin) to mimic the conditions AuSHINs experience during delivery to both cell types. The nanoparticle size distribution and the polydispersity index (PdI) were determined using DLS. As shown in Table 1, no changes in the hydrodynamic diameter of AuSHINs were observed in ultrapure water or 10x diluted PBS, with an average of about 28 nm, confirming their colloidal stability. In contrast, experiments in complete DMEM and RPMI culture media showed an increase in the hydrodynamic diameter to ca. 50 and 45 nm, respectively, as well as an increase in PdI from 0.1 to 0.3. For comparison, complete cell culture media DMEM and RPMI alone have hydrodynamic diameters of (15.1 ± 0.7) nm (PdI = 0.4) and (15.2 ± 0.5) nm (PdI = 0.4), respectively. Bovine serum albumin (BSA), the major component of fetal bovine serum (FBS), has been shown to adsorb onto colloidal silica.^38^ Therefore, one can hypothesize that FBS proteins adsorb onto the AuSHINs, forming a corona,^38−40^ suggesting that there are no AuSHINs clusters in the cell medium. The colloidal stability observed for AuSHINs in the different media, including the culture medium containing serum, was attributed to the silica coating. This coating stabilizes the nanoparticles while preserving their biocompatibility and hydrophilicity, without significantly altering the original plasmonic properties of the AuNPs, an essential factor for PTT effectiveness.^13,14,41,42^

**Table 1:** Average Size, Determined by DLS (±Standard Deviation) and Polydispersity Index (PDI) of AuSHINs^a^

Dispersant	Ultrapure water	PBS 10x diluted	cDMEM	cRPMI
**DLS (nm)**	28.0 ± 0.3	28.6 ± 0.6	50.2 ± 0.7	44.8 ± 0.3
**PdI**	0.1	0.1	0.3	0.3

a The AuSHINs size distribution was determined using DLS in ultrapure water, in 10× diluted PBS, and in complete RPMI as well as in DMEM cell culture media (containing 10% FBS + 1% penicillin) at 25 °C (cRPMI and cDMEM). The final AuSHINs concentration was 1 × 10^12^ NPs/mL.

### AuSHINs–ATTO 647N Incorporation

**Figure 2:**
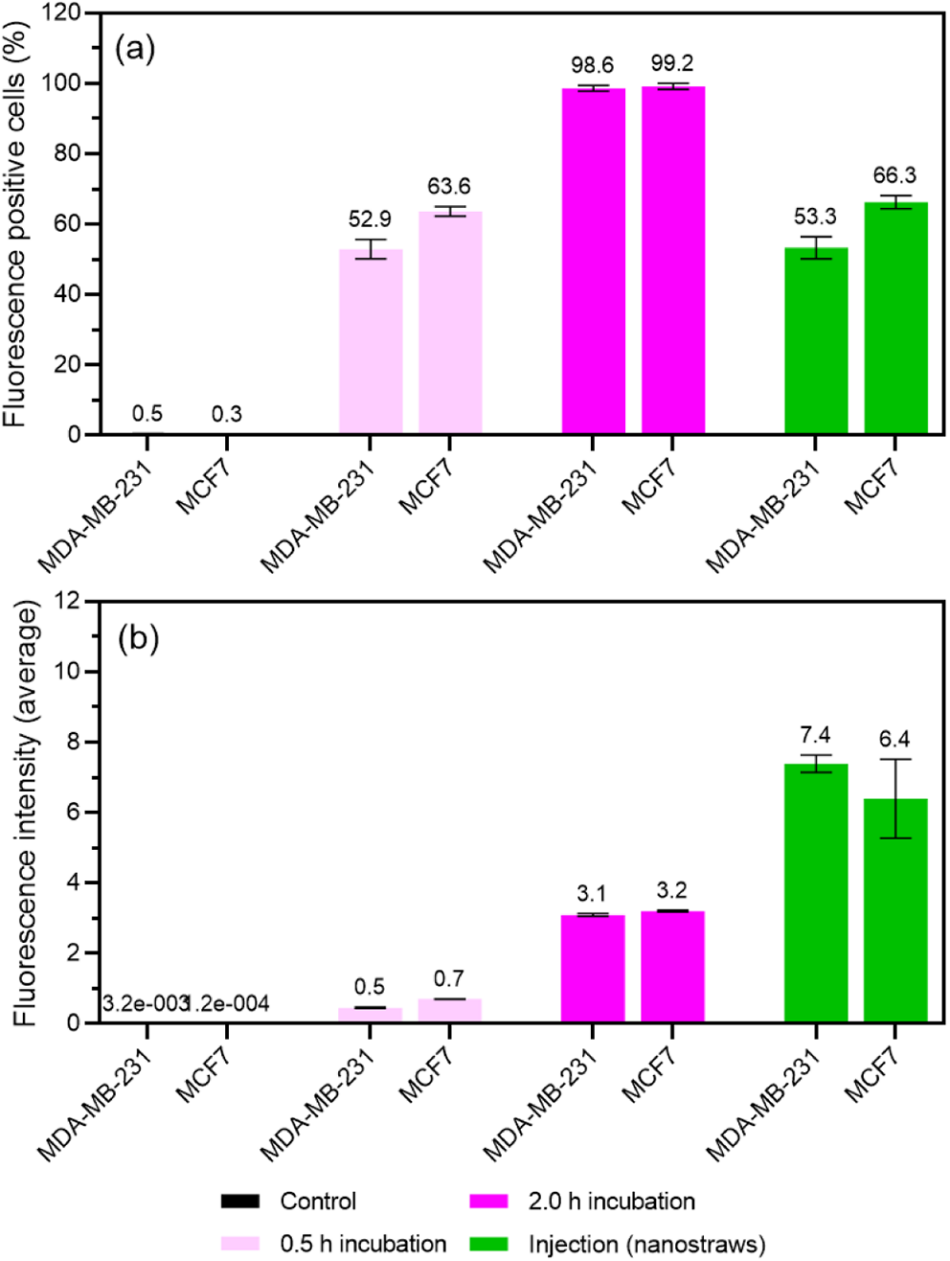
Incorporation of AuSHINs–ATTO 647N into MDA-MB-231 and MCF7 cells via nanostraw-assisted injection (injection (nanostraws)), 0.5 h incubation and 2.0 h incubation represented by (a) percentage of fluorescence positive cells (mean value ± standard deviation) and (b) average fluorescence intensity (*I*) (mean value ± standard deviation), estimated from the equation , where *A* is the mean fluorescence signal of the positive cells, *n*_fluorescence_ is the number of fluorescence positive cells, and *n*_total_ is the total number of cells. Untreated cells were used as a control (cells cultured in culture medium for 2.0 h).

Cells incubated with AuSHINs/AuSHINs–ATTO 647N in cell medium for 0.5 and 2.0 h, or injected with AuSHINs using nanostraws, are referred to as “cells exposed to AuSHINs/AuSHINs–ATTO 647N”, and the process is called “exposure” thereafter. Figure 2 shows the AuSHINs–ATTO 647N cellular incorporation into MDA-MB-231 and MCF7 cells after exposure, determined using flow cytometry. Figure 2a shows the percentage of cells with incorporated AuSHINs–ATTO 647N, and Figure 2b shows the ATTO 647N average fluorescence intensity over the whole cell population, which is proportional to the number of internalized nanoparticles. We verified that cell viability was not affected by AuSHINs–ATTO 647N incorporation (Figure S1). After 0.5 h of incubation and nanostraw injection, ≳50% of cells had incorporated AuSHINs–ATTO 647N, and after 2.0 h of incubation, nearly all the cells had incorporated AuSHINs–ATTO 647N (Figure 2a). Notably, the AuSHINs–ATTO 647N average fluorescence intensity after nanostraw-assisted injection is more than 10-fold higher than the signal after 0.5 h of incubation and around 2-fold higher than the signal after 2.0 h of incubation (Figure 2b). When considering only cells with incorporated AuSHINs–ATTO 647N, this translates into ≈10 times and ≈4 times more internalized AuSHINs–ATTO 647N when nanostraw injection is used compared to 0.5 and 2.0 h of incubation, respectively. Therefore, despite not delivering nanoparticles to all cells, nanostraw injection can deliver substantially higher numbers of AuSHINs–ATTO 647N into MDA-MB-231 and MCF7 cells. The fact that not all cells are injected with AuSHINs–ATTO 647N using nanostraws can possibly be explained by the random distribution of the nanostraws, resulting in cells interfacing with different numbers of nanostraws. It could also be explained by the cell position with respect to the top wire electrode, which is inserted in the cell medium. Indeed, cells further away from the electrode experience a lower electric field, which can affect the transport of AuSHINs–ATTO 647N to the cytosol. This could be improved in future studies by using arrays of nanostraws and using a flat geometry top electrode.

### PTT Effects of AuSHINs on Human Breast Cancer Cells

**Figure 3:**
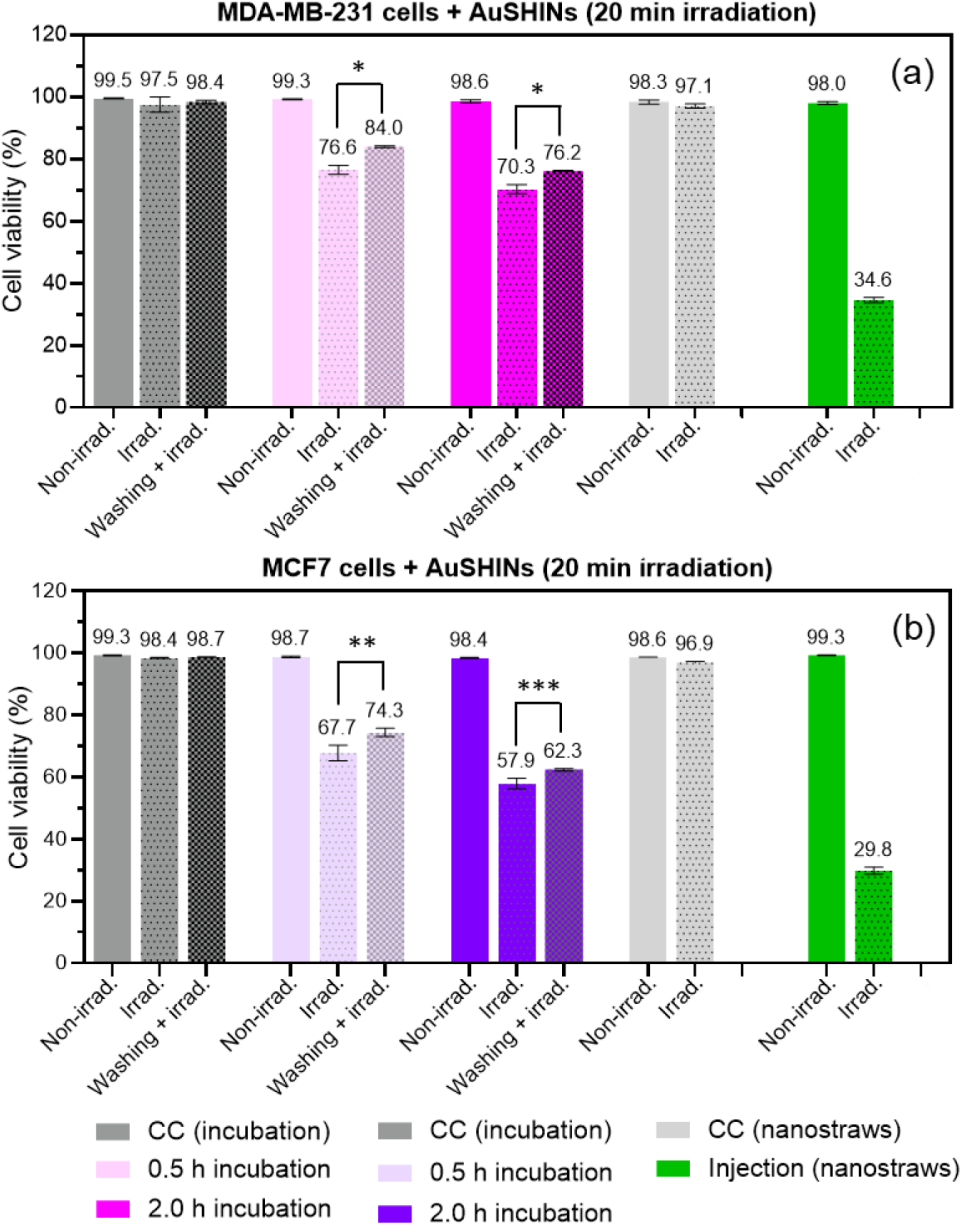
Comparison of AuSHINs photothermal efficiency on (a) MDA-MB-231 and (b) MCF7 cells using 0.5 h incubation, 2.0 h incubation, and nanostraw-assisted injection (injection (nanostraws)) as delivery method. After AuSHINs delivery, cells were irradiated for 20 min at 525 nm ((4.8 ± 0.2) mW/cm^2^, irrad). CC corresponds to cellular controls (without AuSHINs), obtained in the dark (non-irrad.), under irradiation (irrad.), and under irradiation after washing (washing + irrad.). CC (nanostraws) correspond to cells which were injected with only PBS (without AuSHINs), in the dark (Non-irrad.) and under irradiation (irrad.). *p < 0.0001, ** *p* = 0.0002 and *** *p* = 0.005 (multiple t-tests, Bonferroni) in relation to the washing + irrad. populations when irrad. populations are compared (mean value ± standard deviation).

We employed flow cytometry to investigate whether the differences in the number of internalized AuSHINs, when using nanostraw injection *vs* incubation, had effects on the cells. After exposure, cells were irradiated at 525 nm for 20 min (power density = (4.8 ± 0.2) mW/cm^2^). Control cells were subjected to the same treatment without irradiation. For incubation, the effect of washing was also investigated by removing the colloidal suspension from the cell culture after the incubation period and replacing it with fresh culture medium before irradiation. This procedure ensures that only incorporated or membrane-attached AuSHINs remained in the cell culture, therefore making the PTT efficiency between the two different methodologies (injection *vs* incubation) more amenable to comparison. Irradiation alone or AuSHINs alone did not have any negative effect on cell viability, as shown in Figure 3. This demonstrates the nontoxic nature of AuSHINs and the absence of phototoxicity arising from light exposure. In contrast, when incubated with AuSHINs for 0.5 and 2.0 h and exposed to light, MDA-MB-231 cells had decreased viability to 76.6% and 70.3%, respectively (Figure 3a). This small difference in viability is surprising because twice as many cells contain AuSHINs–ATTO 647N, and the average number of AuSHINs–ATTO 647N in cells is higher after 2.0 h than after 0.5 h of incubation (Figure 2). A substantially lower cell viability should be expected after 2.0 h of incubation. This suggests that cells that take up AuSHINs at the beginning of the incubation period are more sensitive to PTT. When AuSHINs were removed from the cell culture by washing before irradiation, the cell viability was higher than for non-washed irradiated cells, with 84% and 76.2% viable cells for 0.5 and 2.0 h of incubation, respectively. Therefore, AuSHINs in the surrounding medium also play a role in cytotoxicity. When using nanostraw injection, on the other hand, MDA-MB-231 cells were significantly affected by irradiation, and the viability was reduced to 34.6%, which means that all cells containing nanostraw-injected AuSHINs died during irradiation (see Figure 2). A similar behavior was observed for MCF7 cells, in which 0.5 and 2.0 h of incubation with AuSHINs led to a decrease in cell viability to 67.7% and 57.9 %, respectively, and to 74.3% and 62.3% if washing was performed before irradiation (Figure 3b). As seen for MDA-MB-231 cells, all MCF7 cells containing AuSHINs after nanostraw injection died during irradiation, leading to a cell viability of 29.8%.

The results highlight the superiority of nanostraw-assisted injection for delivering PTT nanoparticles, which may be attributed to the higher number of nanoparticles delivered compared to those obtained using incubation. Interestingly, the PTT effects of AuSHINs on MCF7 cells were more pronounced than those on MDA-MB-231 cells, regardless of the delivery method. MDA cells are derived from metastatic breast cancer patients and are classified as “triple-negative” cancer cells. Triple-negative cancer cells do not express the receptors for estrogen, progesterone, and human epidermal growth factor 2. In addition, they were more aggressive and difficult to treat than other cancer subtypes,^43,44^ which might explain the greater resistance of MDA-MB-231 cells to PTT treatment.

**Figure 4:**
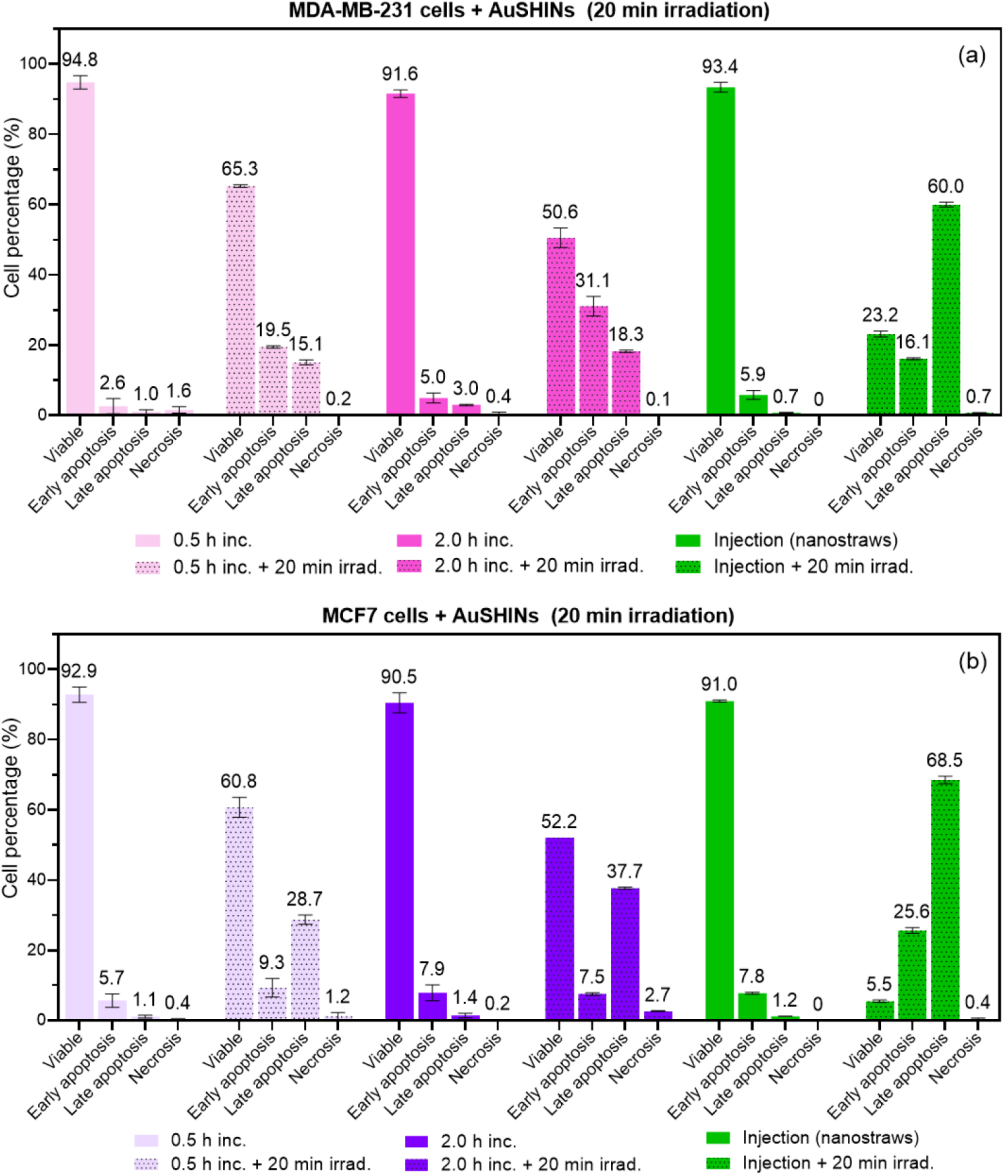
Percentage of (a) MDA-MB-231 and (b) MCF7 cells stained as viable, in early apoptosis, in late apoptosis, or necrosis, to assess cell death pathways induced by AuSHINs delivered through incubation (0.5 h inc. and 2.0 h inc.) or nanostraw-assisted injection (injection (nanostraws)), followed by 20 min of irradiation at 525 nm (+20 min irrad., (4.8 ± 0.2) mW/cm^2^). Cellular controls showed over 92% viability, independent of the delivery method, irradiation, or cell type, as exhibited in Figure S2. Data are presented as the mean value ± standard deviation.

To determine the death pathways induced by PTT, flow cytometry was combined with cellular staining protocols for apoptosis and necrosis. Figure 4 indicates that apoptosis is the main cell death pathway for both the irradiated cell types as well as for both AuSHIN delivery methods. Apoptosis is a highly regulated and ordered process during which cellular contents are confined within membrane-bound vesicles. These vesicles are phagocytosed and removed by immune cells without triggering inflammatory processes, in contrast to when cells die via necrosis. Therefore, by inducing apoptosis but not necrosis, AuSHIN PTT prevents inflammation and damage to nearby cells and tissues.^45,46^ Irradiated MCF7 cells are in a more advanced stage of apoptosis compared with irradiated MDA-MB-231 cells, which is reflected by the decrease in viability for this cell type (Figure 3). As stated above, this might be explained by the higher resistance of MDA-MB-231 cells to therapeutic treatments compared to MCF7 cells.^43,44^

**Figure 5:**
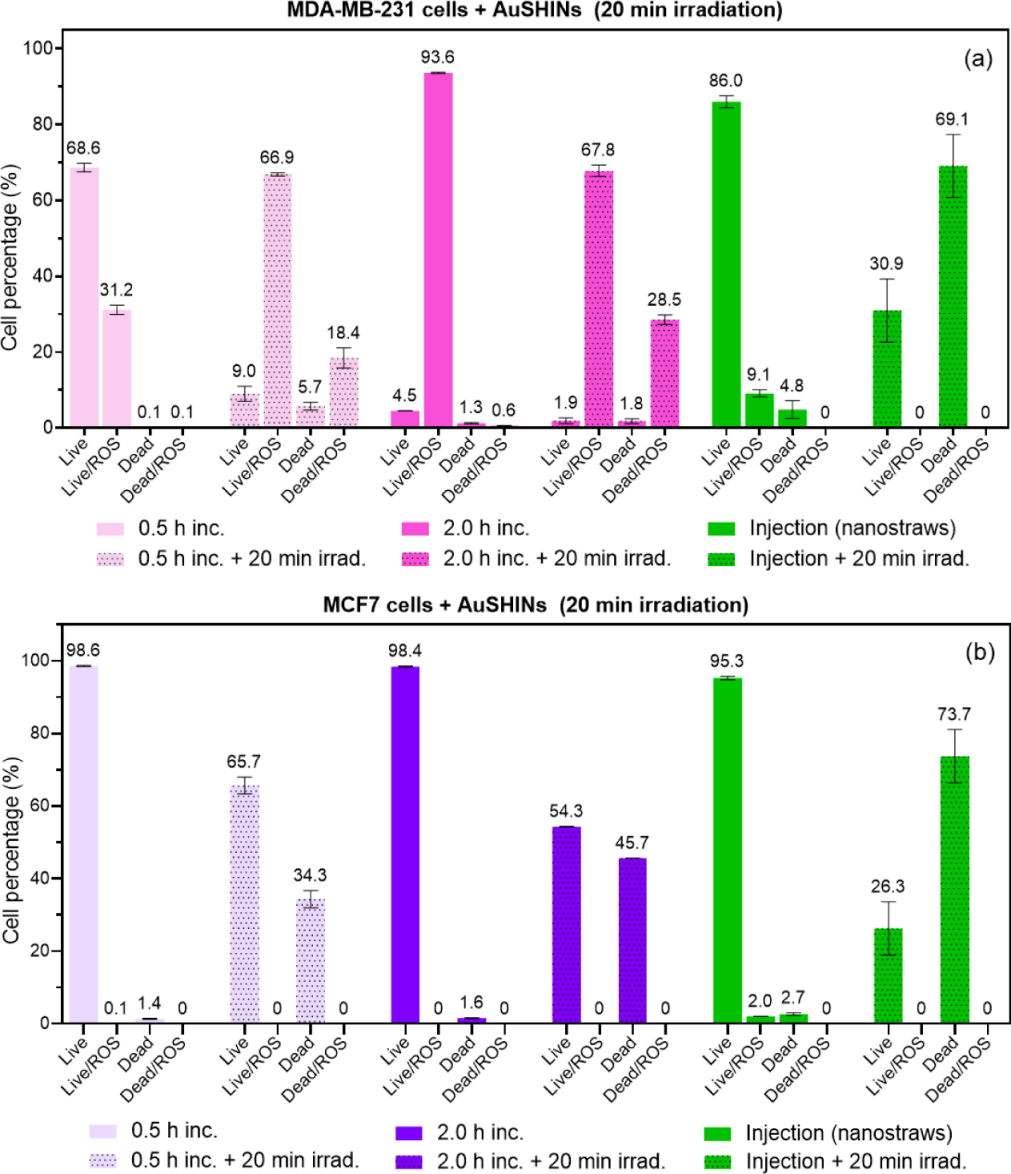
Percentage of (a) MDA-MB-231 and (b) MCF7 cells categorized as live cells (Live), live cells with ROS (Live/ROS), dead cells (Dead), and dead cells with ROS (Dead/ROS), to analyze ROS generation induced by AuSHINs delivered via incubation (0.5 and 2.0 h inc.) or nanostraw-assisted injection (Injection (nanostraws)), followed by 20 min of irradiation at 525 nm (+20 min irrad.). Cellular controls are presented in Figure S3. Data are shown as mean values ± the standard deviation.

The presence of reactive oxygen species (ROS) generated in the MDA-MB-231 and MCF7 cells was also investigated. ROS are chemically reactive molecules that contain oxygen, including superoxide anion radicals (O_2_^–^), hydrogen peroxide (H_2_O_2_), hydroxyl radicals (•OH), and singlet oxygen (^1^O_2_).^47,48^ While ROS play important roles in cell signaling and immune function, excessive ROS levels prompt oxidative reactions, damaging biomolecules and organelle structures in processes such as protein oxidative carbonylation, lipid peroxidation, DNA/RNA breakage, and membrane structure destruction.^49^ In PTT, photoexcited AuSHINs can decay into hot electrons and sensitize molecular oxygen (^3^O_2_), generating ROS via energy and/or electron transfer.^11,12,50^ Besides hyperthermia, the ROS eventually generated in PTT can also trigger tumoral cell death.^11,12,14,16,51,52^ The experiments were conducted using flow cytometry and a ROS-associated live/dead cell labeling kit. The results are shown in Figure 5, while the cellular controls are displayed in Figure S3. No ROS was detected in any MCF7 cells, except before irradiation, when AuSHINs were injected via nanostraws, where 2.0% of the cells were alive and contained ROS (Figure 5b). A possible explanation for the presence of ROS in 2.0 % of MCF7 cells is the rapid intracellular delivery of AuSHINs via nanostraw-assisted injection, possibly also coupled to the co-localization of AuSHINs with the ER. However, this phenomenon is transient since MCF7 cells show no detectable ROS after 20 min of irradiation (Figure 5b). Concerning MDA-MB-231 cells incubated with AuSHINs for 0.5 h, 68.6% of the cells were live cells without ROS, and 31.2% were live cells containing ROS (Figure 5a). In contrast, for 2.0 h of incubation with AuSHINs, only 4.5% of the cells were live cells without ROS content, whereas 93% of the cells were alive and contained ROS. For cellular controls, only 1.3% of cells contained ROS (Figure S3a). Since only half of the cells contain AuSHINs after 0.5 h of incubation and almost all cells have incorporated AuSHINs after 2.0 h, on average double the amount of incorporated AuSHINs (Figure 2a), the results of MDA-MB-231 cells incubated with AuSHINs suggest that ROS generation scales with the number of internalized nanoparticles. Although almost all cells exhibited ROS generation after 2.0 h of incubation, the percentage of dead cells was very low (1.3%), indicating no acute cytotoxicity effect caused by the presence of AuSHINs-induced ROS, at least at this time point. The large difference in ROS content between 0.5 and 2.0 h of incubation time, combined with the similar proportion of dead cells after irradiation, suggests that cell death of irradiated MDA-MB-231 cells incubated with AuSHINs is not solely induced by ROS. However, one cannot rule out that oxidative stress triggered by ROS facilitates cell death in the PTT treatment. Indeed, for 0.5 h of incubation, nanoparticles are still internalized during the 20 min of irradiation, which could explain the increase in total ROS-positive cells after irradiation compared to the same cells before irradiation (from 31.2% to 66.9% live cells with ROS and from 0.1% to 18.4% dead cells with ROS). One can hypothesize that nanoparticle internalization saturates after 2.0 h of incubation, which would explain the constant amount of ROS-positive cells before and after irradiation.

Compared to incubation, AuSHINs nanostraw injection resulted in only 9.1% of MDA-MB-231 live cells containing ROS. After irradiation, 30.9% of the cells are alive and 69.1% are dead, without any ROS content (Figure 5a). Therefore, we conclude that nanostraw injection does not result in ROS generation, in contrast to the case of incubation. Such a striking difference can possibly be explained by the different delivery processes and AuSHINs’ location in the cells. Indeed, nanostraw-assisted injection is much faster than incubation. Although 9.1% of the cells were ROS-positive after AuSHINs injection in MDA-MB-231 cells, no ROS were detected in any cells after irradiation. Hence, cells were capable of recovering from the oxidative stress generated by the injection delivery before being irradiated, and a direct effect of ROS in the cell death mechanism can be excluded.

### Distribution of AuSHINs–ATTO 647N in Cells

The larger PTT effects for nanostraw injection can be attributed to the localization of AuSHINs, which we assessed using confocal fluorescence microscopy. MDA-MB-231 and MCF7 cells containing AuSHINs–ATTO 647N (red), delivered using the nanostraw-assisted injection or 2.0-h incubation method, were labeled with either LysoTracker (green) or ER-Tracker (green), and Hoechst 33342 (blue). Additional confocal fluorescence microscopy images of MDA-MB-231 and MCF7 cells containing AuSHINs–ATTO 647N, delivered via incubation or nanostraw-assisted injection, are provided in Figures S4 and S5.

**Figure 6:**
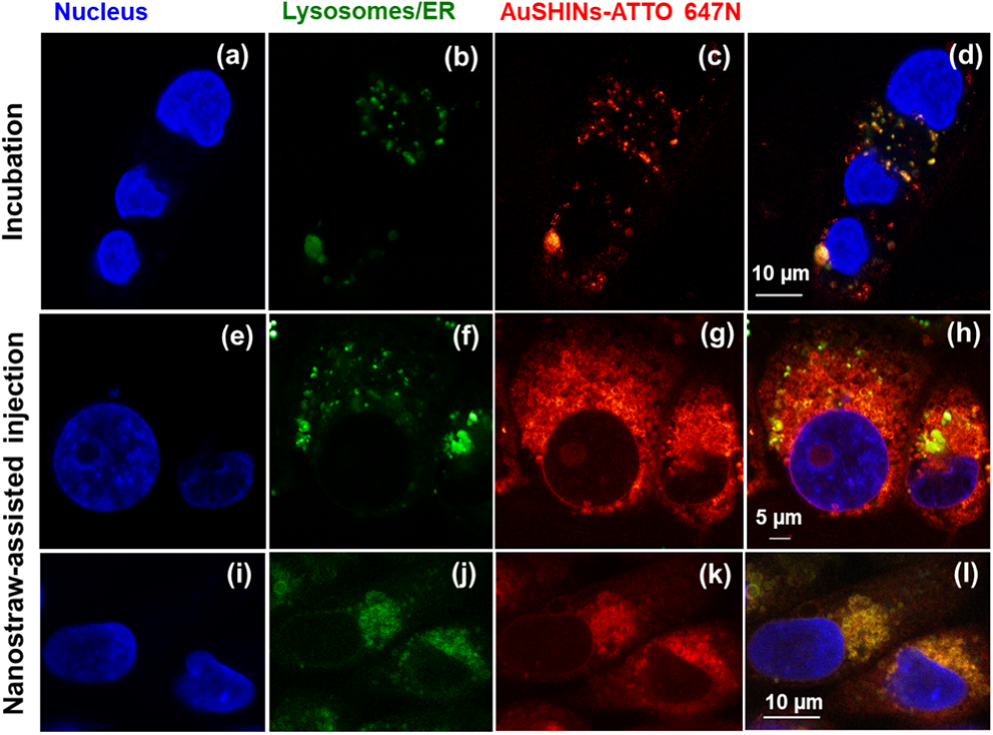
AuSHINs–ATTO 647N fate in MDA-MB-231 cells after (a)–(d) 2.0 h of incubation and (e)–(l) nanostraw-assisted injection imaged by confocal fluorescence microscopy. AuSHINs–ATTO 647N (red), lysosomes (green in (b), (d), (f), and (h), stained with Lysotracker), ER (green in (j) and (l), stained with ER Tracker) and nucleus (blue, labeled with Hoechst 33342).

**Figure 7:**
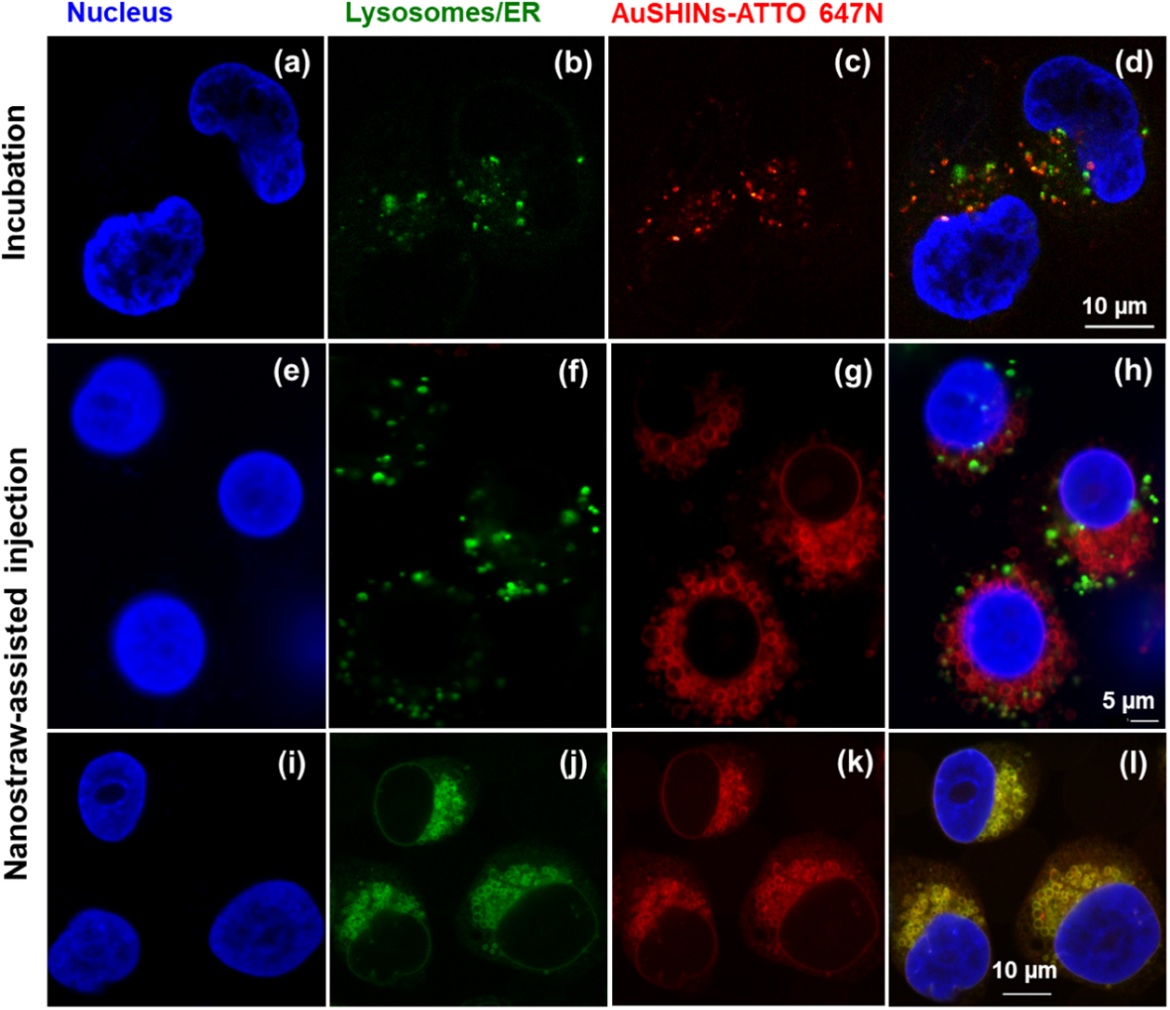
AuSHINs–ATTO 647N fate in MCF7 cells after (a)–(d) 2.0 h of incubation and (e)–(l) nanostraw-assisted injection imaged by confocal fluorescence microscopy. AuSHINs–ATTO 647N (in red), lysosomes (green in (b), (d), (f), and (h), stained with Lysotracker), ER (green in (j) and (l), stained with ERTracker), and nucleus (blue, labeled with Hoechst 33342).

With 2.0 h of incubation, the majority of internalized AuSHINs–ATTO 647N is colocalized in the lysosomes (Figures 6a,b and 7a,b). Therefore, the nanoparticles seem to be internalized via endocytosis and do not avoid endosomes, as has been reported for other nanoparticles.^53−59^ This lysosomal entrapment can be expected, since the nanoparticles are negatively charged (ζ-potential = −33.6 mV) and are not best suited to fuse with the negatively charged endosome membrane, in contrast to ionizable lipid nanoparticles.^60^ For nanostraw-assisted injection, however, the majority of AuSHINs–ATTO 647N was found outside the lysosomes (Figures 6c,d and 7c,d), instead accumulating in the ER (Figures 6e,f and 7e,f). Most of the AuSHINs–ATTO 647N injected via nanostraws avoid lysosomal entrapment and are delivered directly into the cytosol, similarly to the injection of fluorescent nanodiamonds into cells.^24^ In addition to the higher number of delivered nanoparticles, the accumulation of nanoparticles in the ER may explain the superiority of PTT when using nanostraws compared to incubation. Therapeutic agents that can enter cells and target the ER are crucial for achieving satisfactory outcomes in cancer treatment,^17^ since cancer is closely related to ER stress.^61−63^ In the tumor microenvironment, high levels of stress are required to maintain rapid proliferation and metastasis of tumor cells. In other words, ER stress enhances the tumor cells’ ability to adapt to unfavorable environments and promotes malignant progression.^63,64^ Therefore, targeting the ER might reduce the protective effects of ER stress on cancer cells.

In summary, the nanostraw injection method is promising for PTT nanoparticle delivery by increasing the number of internalized nanoparticles, circumventing lysosomal entrapment, which is a considerable challenge in therapeutic development,^24,65^ and by targeting the ER. Though it is not straightforward to translate the nanostraw technology *in vivo*, the results presented here highlight the importance of the nanoparticle cellular end point in PTT efficiency.

## Conclusion

Nanostraw-assisted injection has proven to be a more efficient method for delivering AuSHINs to MDA-MB-231 and MCF7 breast cancer cells, with a 2-fold increase in nanoparticle uptake compared to incubation. Moreover, after irradiation, all cells containing nanostraw-injected AuSHINs died, compared to only a third when using incubation. For both delivery methods and cell lines, the cell death pathway is apoptosis, thereby avoiding necrosis-induced inflammation and damage to surrounding tissues. Notably, nanostraw injection resulted in a faster onset of apoptosis compared to incubation. Additionally, while no ROS were detected in MCF7 cells under any conditions, ROS generation was observed in irradiated MDA-MB-231 cells following incubation but not after nanostraw injection. Confocal microscopy images showed that nanostraw-injected AuSHINs ended up in the endoplasmic reticulum, whereas traditional incubation primarily resulted in AuSHINs localized in lysosomes. This AuSHINs accumulation at the ER significantly contributes to the enhanced PTT efficiency. In conclusion, the nanostraw-assisted injection method significantly enhances the effectiveness of AuSHINs-mediated PTT by improving both the delivery efficiency and ensuring a more effective intracellular end point for nanoparticles. In general, the results presented here highlight the importance of delivering PTT nanoparticles to the right location in the cells and point to the ER as a relevant end point.

## Experimental Section

### AuSHINs Synthesis and Characterization

Spherical gold nanoparticles (AuNPs) were synthesized and coated with silica, resulting in gold-shell isolated nanoparticles (AuSHINs), according to previous protocols.^16,34^ To evaluate the cellular incorporation and localization of AuSHINs, they were conjugated with a fluorophore, ATTO 647N (MW = 811 g/mol, Merck Life Science), following a well-established methodology.^35,37,66^ Specifically, 128 μL of ATTO 647N aqueous solution (10 μmol/L in ultrapure water) was mixed with 2.5 mL of an AuSHINs colloidal suspension (1 × 10^13^ AuSHINs/mL), under magnetic stirring in the dark at room temperature for 24 h. The protonated amide group of ATTO 647N binds electrostatically to the negatively charged AuSHINs, resulting in AuSHINs–ATTO 647N nanoparticles. The size, shape, and silica shell thickness of the nanoparticles and their colloidal stability were assessed using UV-Vis absorption spectroscopy, scanning electron microscopy (SEM), transmission electron microscopy (TEM), dynamic light scattering (DLS), and zeta potential measurements (ζ). Each measurement was performed at least in triplicate to ensure the reproducibility of the experiments.

The UV-Vis absorption spectra from 200 to 800 nm were obtained with a Nanodrop 2000 Spectrophotometer (Thermo Scientific), which utilizes a xenon flashlight source, with a 2048-element linear silicon CCD array detector, and 5 W power. SEM images (ZEISS Gemini 500) were acquired with an acceleration voltage of 10 kV, an InLens detector, and a sample holder carousel measuring 9 × 10 mm. TEM images (JEOL JEM-1400) were collected with an acceleration voltage of 80 kV and a resolution point of 0.23 nm. DLS and ζ-potential measurements (Zetasizer Nano ZS, Malvern Panalytical Ltd., Malvern, UK) were performed with a 4 mW He–Ne laser (632.8 nm) to determine the hydrodynamic diameter and surface charge of the nanoparticles, respectively. The hydrodynamic diameter (z-average) was extracted by cumulant analysis of the data, and the polydispersity index (PdI) was obtained from cumulant fitting.

### Cell Culture of Human Breast Carcinoma

Human carcinoma cells MDA-MB-231 (human breast adenocarcinoma) and MCF7 (glandular breast carcinoma) were purchased from the European Collection of Authenticated Cell Cultures (ECACC via Sigma-Aldrich/Merck). Thawed cells were seeded at a density of ca. 5000 cells/cm^2^ in T25 cell culture flasks (Nunc, 156367, Thermo Fisher Scientific) and cultured in a cell culture medium at 37 °C under 5% CO_2_ atmosphere, supplemented with 10% fetal bovine serum (FBS, Sigma-Aldrich) and 1% penicillin-streptomycin (P4333, BioReagent, Sigma-Aldrich). The cell medium was Dulbecco′s Modified Eagle′s Medium (DMEM; Gibco, Thermo Fisher) for MDA-MB-231 cells and RPMI 1640 (Gibco, Thermo Fisher) for MCF7 cells. Cells were passaged upon reaching approximately 80–90% confluency, as reported for breast cancer cells and other cancer cell types.^12,16^

### Fabrication of Nanostraws

Nanostraws for AuSHINs delivery were fabricated from track-etched polycarbonate (PC) membranes (it4ip, Belgium), with an inner diameter of 200 nm, a thickness of 25 μm, and a pore density of 2E7 cm^–2^, following a well-established protocol.^24,25^ Using atomic layer deposition (ALD, Savannah, Cambridge Nanotech), the PC membranes were coated with ≈12 nm of alumina. For this, the PC membranes were exposed to 130 cycles of alternating pulses of trimethylaluminum and H_2_O (0.15-s-long pulses with a 30-s waiting time in between) at 90 °C. Inductively coupled plasma and reactive ion etching (ICP-RIE, APEX SLR Advanced Vacuum Systems AB) was performed in two steps to obtain nanostraws. To achieve sufficient heat transfer during etching, an antistatic gun (Zerostat, VWR) was used to make the coated membranes adhere to a 4 in. silicon wafer. The first ICP-RIE step removed the alumina from the horizontal surfaces of the PC membrane by using argon at 40 sccm for 2.5 min (RIE power set to 60 W and ICP power set to 400 W). The second ICP-RIE step ≈1 μm of PC by using SF_6_ at 5 sccm and O_2_ at 45 sccm for ≈1.5 min (RIE and ICP power set to 50 W and 400 W, respectively). Helium cooling was maintained at a flow rate of 5 sccm for both etching processes. The PC membranes were then imaged using SEM (LEO Gemini 1560, LEO Electron Microscopy, Inc.), after being mounted on an SEM stub with carbon tape and sputter-coated with 5–10 nm of Pt:Pd (80:20) or Ir (Q150T ES sputter coater, Quorum Technologies). The height and inner diameter were measured to ∼1 μm and 160 nm ± 10 nm, respectively.

Nanostraw devices for cellular injections were prepared by attaching the nanostraw substrates to polymer cylinders (10/7 mm in outer/inner diameter and 10 mm in height) using biocompatible double-sided tape (3M 8153LE (300LSE) double-lined Adhesive Transfer Tape). The nanostraws were facing the inside of the cylinder to have contact with the cells. The devices were sterilized for 2 min in UV-light prior to cell seeding.

### AuSHINs Delivery Through Incubation

Cells were seeded in 96-well plates at a density of 5 × 10^4^ cells/well and cultured for 16 h. They were subsequently incubated with 200 μL of AuSHINs (1 × 10^12^ NPs/mL) in culture media for 0.5 and 2.0 h at 37 °C under 5% CO_2_ atmosphere, equivalent to an AuSHINs/cell ratio of 4 × 10.^6^ AuSHINs (1 × 10^12^ NPs/mL in culture media) were prepared by diluting the stock solution (1 × 10^13^ NPs/mL in ultrapure water) in culture media. Following the incubation period, cells were exposed to 525 nm light using a green LED at a power density of (4.8±0.2) mW/cm^2^ for 20 min to induce photoactivation and heating. Cellular controls (CC) correspond to cells treated the same way but not exposed to AuSHINs or light. Light controls (LC) correspond to CC exposed to 20 min of light irradiation at the same power density.

### AuSHINs Delivery via Nanostraws-Assisted Injection

**Figure 8:**
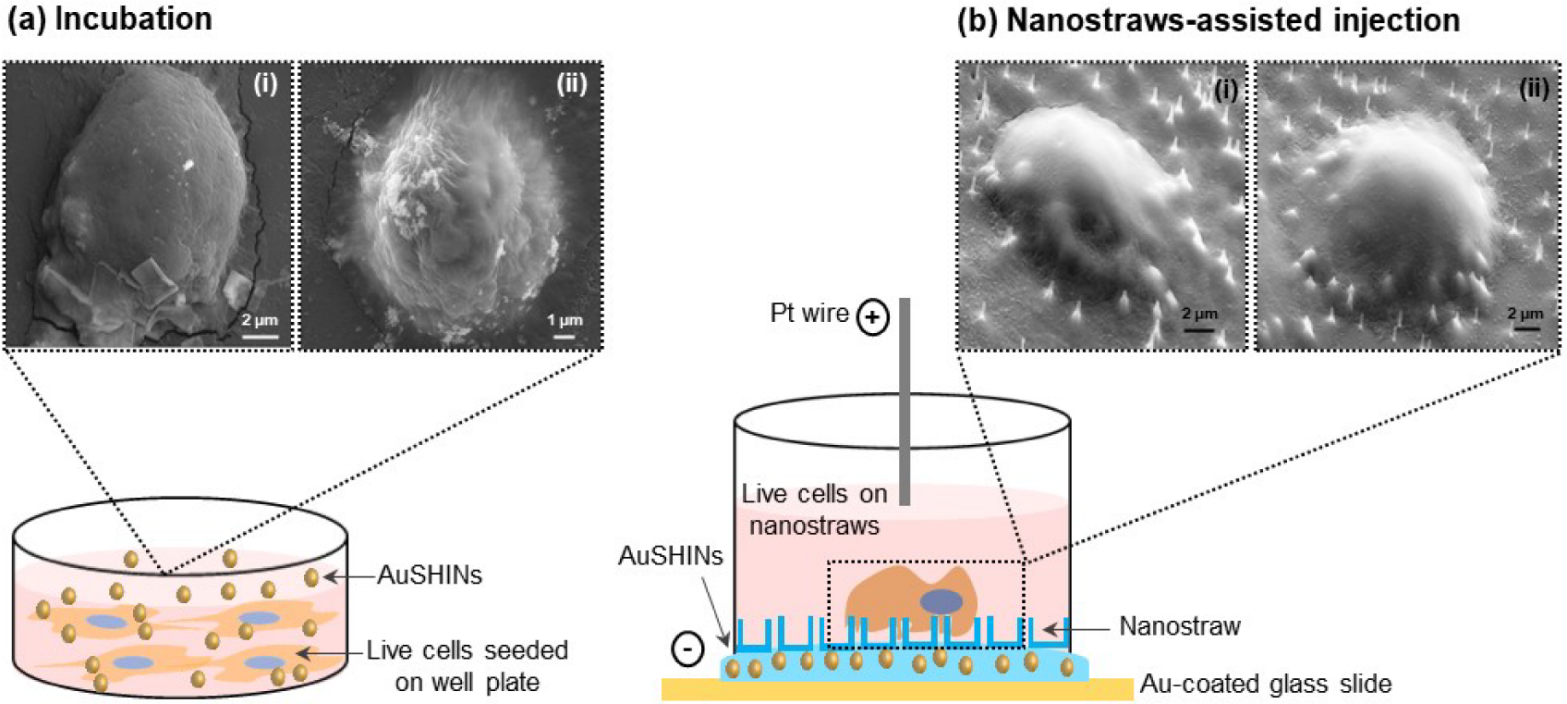
(a) AuSHINs delivered by incubation and (b) intracellular delivery of AuSHINs via nanostraw-assisted injection. The illustrations are not to scale. The insets show SEM images for (i) MDA-MB-231 and (ii) MCF7 cells. The SEM stage tilt was 0° for (a) incubation and 45° for (b) nanostraw-assisted injection.

Cells in suspension were seeded in cylinders containing the culture medium onto the nanostraws (2 × 10^4^ cells/cylinder) and centrifuged at 200 RCF for 1 min. The nanostraw device was placed onto a 10 μL drop of AuSHINs colloidal suspension (1 × 10^12^ NPs/mL, in 10× diluted Phosphate Buffered Saline) (PBS, Gibco, Thermo Fisher)), corresponding to an AuSHINs/cell ratio of 5 × 10^5^, and deposited on a gold-coated glass slide, acting as an electrode. A second electrode (platinum wire) was inserted into the cell medium on top of the cells and nanostraws. Electrical pulses were applied between the electrodes (pulse generator TGP110, Aim and Thurlby Thandar Instruments, Huntingdon, UK) with a signal amplifier (WMA-300, Falco Systems BV, Katwijk Zee, The Netherlands). Two 40-s-long trains of square electrical pulses (28 V, frequency of 40 Hz, pulse duration of 200 μs) were applied between the electrodes with a 1-min waiting time in between. After the injection, cells were pipetted off the nanostraw substrate and transferred to a 96-well plate, where they were exposed to 525 nm using a green LED source for 20 min to induce photothermal effects. The power of the light was measured using a powermeter (Melles Griot 13PEM001) at 2.5 cm from the LED panel, which is the distance at which the cells are placed, and divided by the sensor area to obtain the power density, in this case, (4.8 ± 0.2) mW/cm^2^. Cellular controls (CC) correspond to cells treated the same way but injected with PBS devoid of AuSHINs. Light controls (LC) correspond to cells seeded on nanostraw substrates, injected with only PBS, and subsequently exposed to 20 min of light irradiation ((4.8 ± 0.2) mW/cm^2^). The schematics of the two methodologies used for delivering AuSHINs are shown in Figure 8.

Note that a direct comparison between incubation and nanostraw delivery is difficult due to the different states of the AuSHINs, delivered in cell medium for incubation and in diluted PBS for nanostraw delivery, but also due to the differences in membrane area exposed to AuSHINs and the varying concentrations of AuSHINs. Using the incubation method, AuSHINs were delivered in cell culture medium because cells may experience stress due to the lack of nutrients, serum, and buffering capacity during the 2 h of incubation. To avoid ambiguity about whether the observed cellular stress was induced by experimental conditions or PTT treatment, we therefore delivered AuSHINs in a culture medium. On the other hand, we have shown in another publication^25^ that using culture medium when delivering cargos with nanostraws results in a low voltage drop over the nanostraws, and therefore low transfection efficiency because of the low electrophoretic force driving the cargo inside the cells. Therefore, for each method, we aimed at maximizing the delivery efficiency. This approach involved incubating in cell medium and injecting using 10x diluted PBS. The point of our study is to demonstrate that, when comparing both methods individually optimized, PTT can possibly be more efficient when using nanostraws than when using incubation.

### SEM Imaging of MDA-MB-231 and MCF7 Cells Exposed to AuSHINs via Incubation and Nanostraw-Assisted Injection

MDA-MB-231 and MCF7 cells were cultured for 24 h in 24-well plates (5 × 10^4^ cells/well) containing small square substrates (1 × 1 cm^2^) cut from T25 cell culture flasks (Nunc, 156367, Thermo Fisher Scientific). The cells were then incubated with AuSHINs for 2.0 h at 37 °C under 5% CO_2_. For nanostraw injection, the cells were cultured on the nanostraw substrate in the cylindrical device (2 × 10^4^ cells/well) for 24 h prior to AuSHINs injection. After AuSHINs exposure via incubation or injection, the cells were fixed by replacing the culture medium with 4% paraformaldehyde (PFA) for 10 min at room temperature. PFA was then removed, and the cells were rinsed with PBS and incubated for an additional 10 min. This washing step with PBS was repeated 3 times. Following cell fixation, the cells were dehydrated by sequential incubation with ethanol solutions (20%, 50%, 70%, 90%, 95%, and 99.5%) for 5 min each. After cell fixation, the culture square substrates containing cells incubated with AuSHINs were removed from the 24-well plates and attached on top of SEM stubs for the sputtering process. For nanostraw injection samples, the substrates were detached from the cylindrical devices and similarly mounted on SEM stubs prior to sputtering.

Sputter coating was performed on the samples using 5 nm of iridium (Quorum Q150T ES, Quorum Technologies Ltd., Laughton, UK) at a deposition rate of 2 nm min^–1^. The culture flask squares and nanostraw substrates were then placed on a 9 × 10 mm sample holder carousel and a universal 45° sample holder, respectively, for SEM imaging (ZEISS Gemini 500 SEM), using an acceleration voltage set to 20 kV and a secondary electron detector.

### Fluorescence Labeling of Live Cells

Cells used in incubation experiments were cultured in glass-bottom Petri dishes for 24 h, whereas cells used for nanostraw injection were seeded on the nanostraw devices and cultured for 18 h before fluorescence labeling and AuSHINs–ATTO 647N exposure. The fluorescence labeling of the live cell organelles was performed as follows: for lysosome staining, cells were incubated in LysoTracker Deep Red (Thermo Fisher, L12492) at 75 nmol/L in cell medium for 2 h at 37 °C and 5% CO_2_ in the incubator. For endoplasmic reticulum (ER) staining, cells were incubated in 1 μmol/L ER Tracker Red (Thermo Fisher, BODIPY TR Glibenclamide) in PBS for 30 min at 37°C and 5% CO_2_ in the dark. Subsequently, the cells in glass-bottom Petri dishes were incubated with AuSHINs–ATTO 647N (1.0 × 10^12^ NPs/mL in culture medium) for 2 h, and the cells on the nanostraws were injected with AuSHINs–ATTO 647N (1.0 × 10^12^ NPs/mL in 10x diluted PBS), as described above. The cell nuclei were subsequently stained with Hoechst 33342 (62249, Thermo Fisher Scientific) by incubating cells for 2 min in 2.5 μg/mL Hoechst in Dulbecco’s Phosphate Buffered Saline (DPBS), without calcium and magnesium (Thermo Fisher), before being washed twice with DPBS and eventually replacing DPBS with cell culture medium.

### Confocal Fluorescence Microscopy Images

The AuSHINs–ATTO 647N cellular uptake and localization were assessed in live cells using confocal fluorescence microscopy. For cells on nanostraws, the nanostraw membrane was gently peeled off from the plastic cylinder and placed upside-down inside a 100 μL drop of cell culture medium on a glass coverslip, ensuring that no pressure was applied to the cells. Incubated cells were imaged directly on the glass bottom of Petri dishes. The images were acquired using a STED microscope system, Abberior 2C STED 775 QUAD Scan (Abberior Instruments GmbH, Goettingen, Germany), equipped with a microscope stage cell incubator (OKOLab) and a 60× Oil objective Nikon CFI Plan Apochromat Lambda (NA 1.40). The cell nucleus signal was imaged using a 405 nm excitation laser and a (450/50) nm emission filter, lysosomes and ER were imaged using a 561 nm excitation laser and a (605/50) nm emission filter, and AuSHINs–ATTO 647N were imaged using a 640 nm excitation laser and a (685/70) nm emission filter. All acquired images were processed and analyzed using the software ImSpector and Fiji. For presenting individual images, the contrast and brightness were automatically adjusted in Fiji for each image. Data preprocessing was performed only when images intended for comparison were acquired using the same microscope settings and exposure times.

### Flow Cytometry Measurements

Flow cytometry measurements were performed to assess AuSHINs–ATTO 647N cellular incorporation, cell death pathways (apoptosis and necrosis), and reactive oxygen species generation (ROS) using different kits and staining protocols, as described below. After AuSHINs exposure, cells were detached from their substrate, collected into microtubes (2 × 10^5^ cells/mL) containing culture medium, and analyzed using flow cytometry (MACSQuant Analyzer 16 flow cytometer, Miltenyi Biotec). The data were analyzed using the software MACSQuantify (version 2.13.3) and plotted with GraphPad Prism 8®, and statistical significance was estimated through analysis of variance (one unpaired multiple t-test). The incorporation of AuSHINs–ATTO 647N in MDA-MB-231 and MCF7 cells was evaluated by assessing the fluorescence signal of ATTO 647N in cells. The cell viability was assessed by staining dead cells with DAPI (Thermo Fisher Scientific, 62248), by adding DAPI (10 μg/mL in PBS) to the microtube before analysis. Cell death pathways were determined using the Dead Cell Apoptosis Kit, combining Annexin V Alexa Fluor 488 and Propidium Iodide (PI) (Thermo Fisher Scientific, V13241), according to the manufacturer’s instructions. Phosphatidylserines are expressed in the outer leaflets of the plasma membrane of cells undergoing apoptosis, which can be specifically stained with Annexin V. Cells in late apoptosis and necrosis exhibit holes in the cell membrane, exposing the nucleus to the membrane-impermeable DNA dye PI.^67,68^ The lack of fluorescence (double-negative) characterizes viable cells, a positive fluorescence signal for Annexin V and negative for PI indicates cells in early apoptosis, cells negative for Annexin V and positive for PI are necrotic, and cells positive for Annexin V and PI are in late apoptosis.^68^

The generation of ROS was evaluated using the CellROX® Deep Red Flow Cytometry Assay Kit (Thermo Fisher Scientific, C10491) with slight modifications to the manufacturer’s protocol. For the ROS generation experiments described here, ROS was detected in the cells using the CellROX Deep Red reagent provided in the kit, while live and dead cells were identified using DAPI, following the same protocol as described above. Cells negative for both DAPI and CellROX Deep Red were considered to be alive without ROS, DAPI-negative/CellROX-positive cells were considered alive with ROS, DAPI-positive/CellROX-negative cells were considered dead without ROS generation, and DAPI-positive/CellROX-positive cells were considered dead with ROS. The ROS positive control was performed by incubating the cells with *tert*-butyl hydroperoxide (TBHP), which induces oxidative stress, prior to the CellROX staining protocol. The negative control for ROS generation was established by incubating the cells with the antioxidant *N*-acetylcysteine (NAC) before carrying out the CellROX protocol.

## Associated Content

## Supporting Information Available

Cell viability, proportion of cells in apoptosis, and proportion of cells containing ROS for MCF7 and MDA-MB-231 cellular controls. Additional fluorescence confocal microscopy images of both cell types exposed to AuSHINs via incubation and nanostraw injection (PDF)
