# Supplementary material for: Enhancing Photothermal Therapy Against Breast Cancer Cells by Modulating the End Point of Gold Shell-Isolated Nanoparticles Using Nanostraw-Assisted Injection
Source: ACS Appl Mater Interfaces. 2025 Apr 29;17(19):27816–28. doi: 10.1021/acsami.5c00084 (PMC12086757; doi:10.1021/acsami.5c00084)
Supplement: Supplementary file 1 — am5c00084_si_001.pdf [file am5c00084_si_001.pdf]

Supporting Information

Enhancing Photothermal Therapy Against Breast Cancer Cells By Modulating The End Point Of Gold Shell-Isolated Nanoparticles Using Nanostraw-Assisted Injection

Sabrina A. Camacho[†, ††], *Pedro H. B. Aoki*[†, ††], *Frida Ekstrand*[††], *Osvaldo N. Oliveira Jr.*[†††], *and Christelle N. Prinz*[††]*

[†]School of Sciences, Humanities and Languages, São Paulo State University (UNESP), Assis, SP, 19806-900, Brazil

[††]Division of Solid-State Physics and NanoLund, Lund University, Lund 221 00, Sweden

[†††]São Carlos Institute of Physics, University of São Paulo (USP), São Carlos, SP, 13566-590, Brazil

*Corresponding author: christelle.prinz@ftf.lth.se

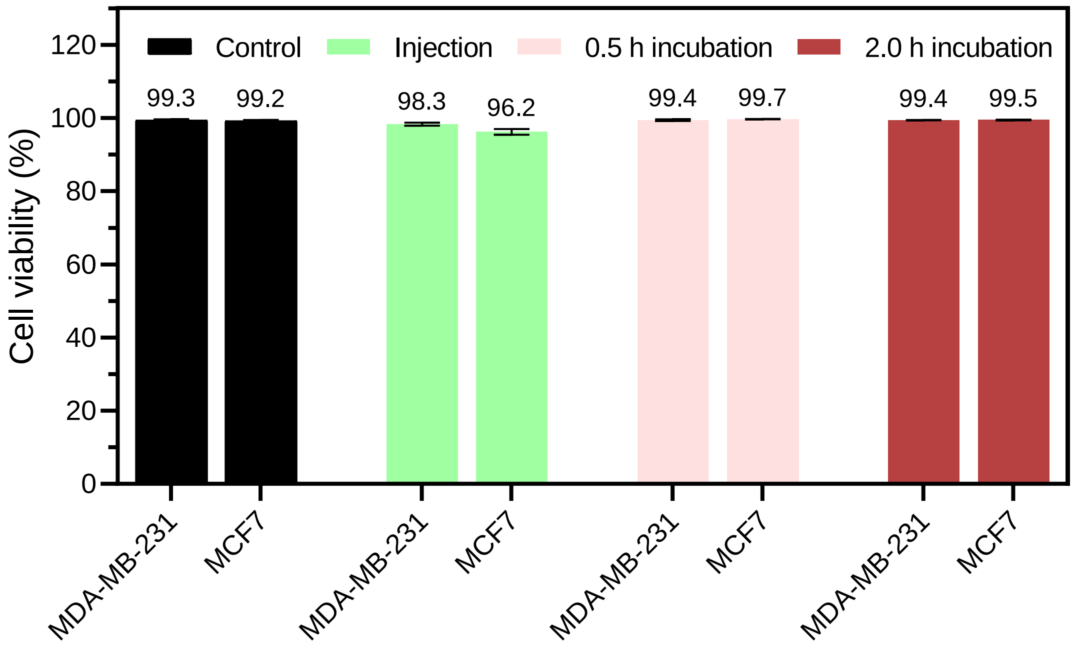

Figure S1. Viability for MDA-MB-231 and MCF7 cells without incorporated AuSHINs–ATTO 647N (control) and after incorporation of AuSHINs–ATTO 647N trough nanostraws-assisted injection (injection) and 0.5 and 2.0 h of incubation.

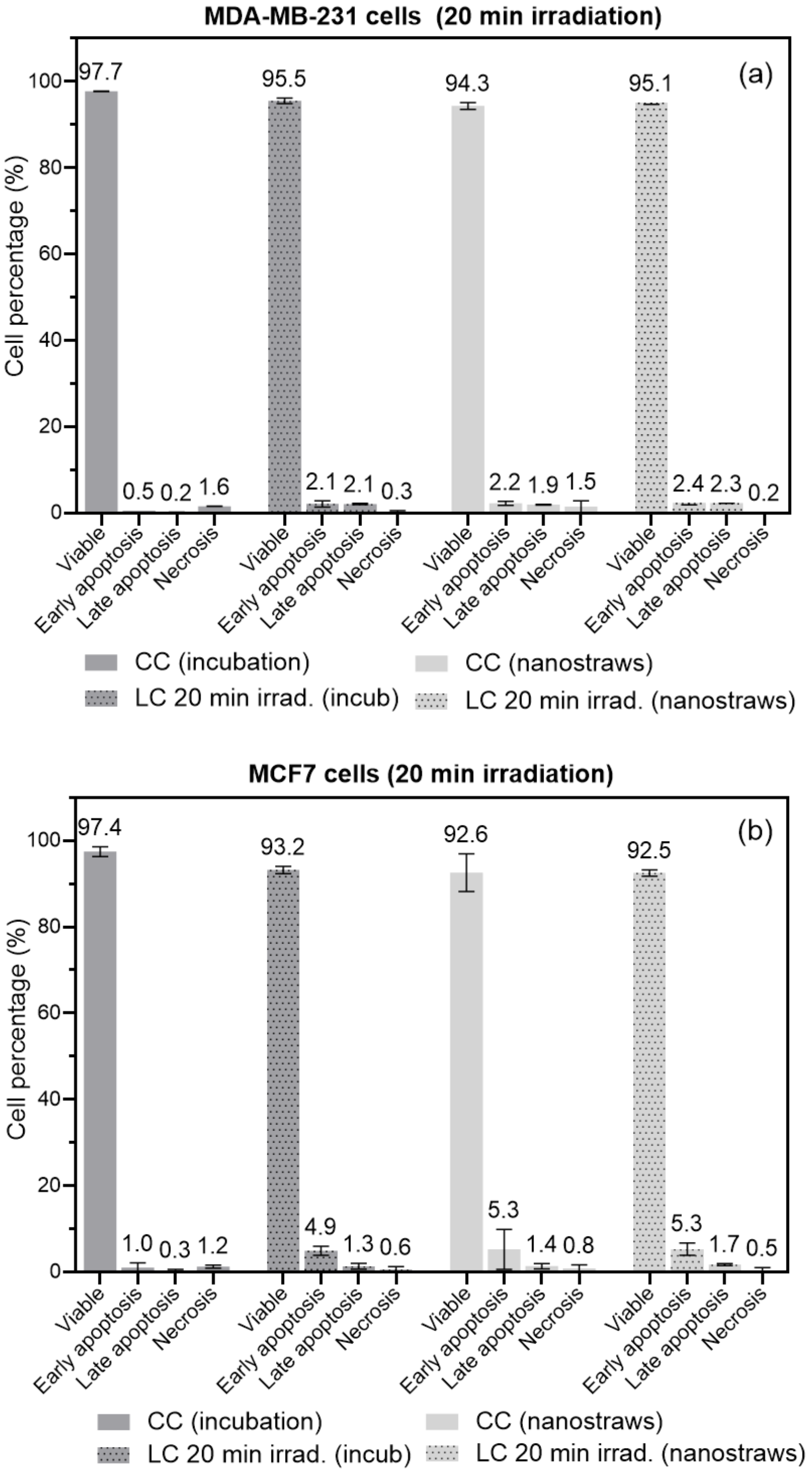

Figure S2. Percentage of cells that are viable, in early apoptosis, in late apoptosis, and necrotic for cellular controls (CC) and light controls (LC) for a) MDA-MB-231 cells and (b) MCF7 cells. Cellular controls are cells seeded in 96-well plates for 16 h not exposed to light irradiation (CC (incubation)) and cells seeded on nanostraw substrate, subjected to an injection of PBS devoid of AuSHINs and not exposed to light irradiation (CC (nanostraws)). LC 20 min irrad. (incub) corresponds to cells seeded on 96-well plates and let adhered for 16 h before exposing them to 20 min irradiation at 525 nm (Power density = (4.8±0.2) mW/cm^2). LC 20 min irrad. (nanostraws) correspond to cells seeded on the nanostraw substrate, subjected to an injection of only PBS, before exposing them to 20 min irradiation at 525 nm.

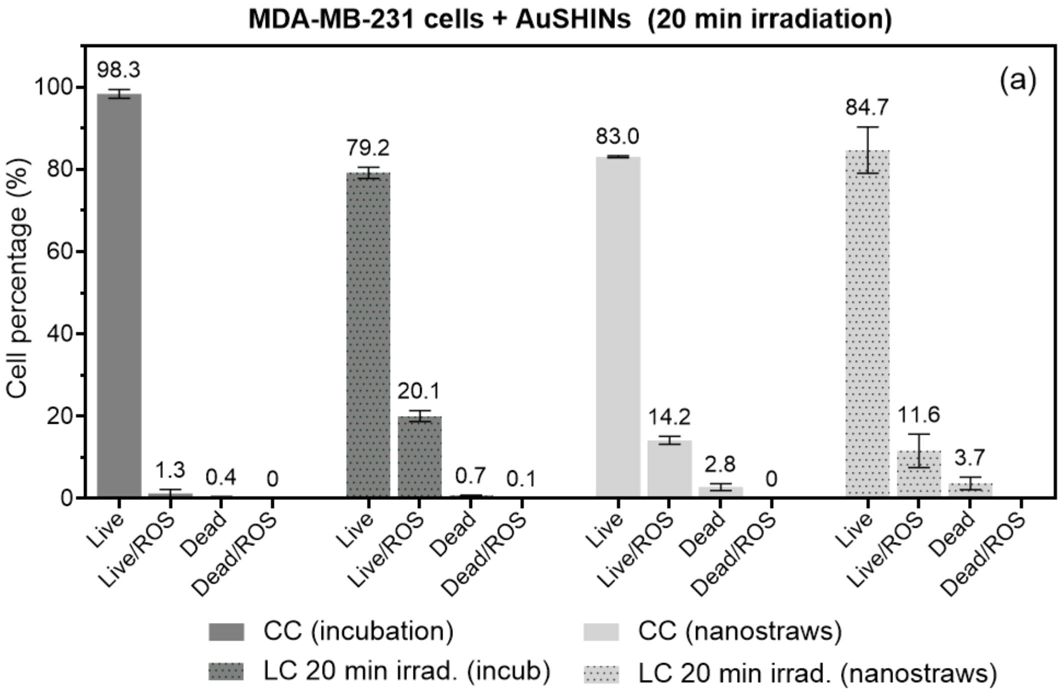

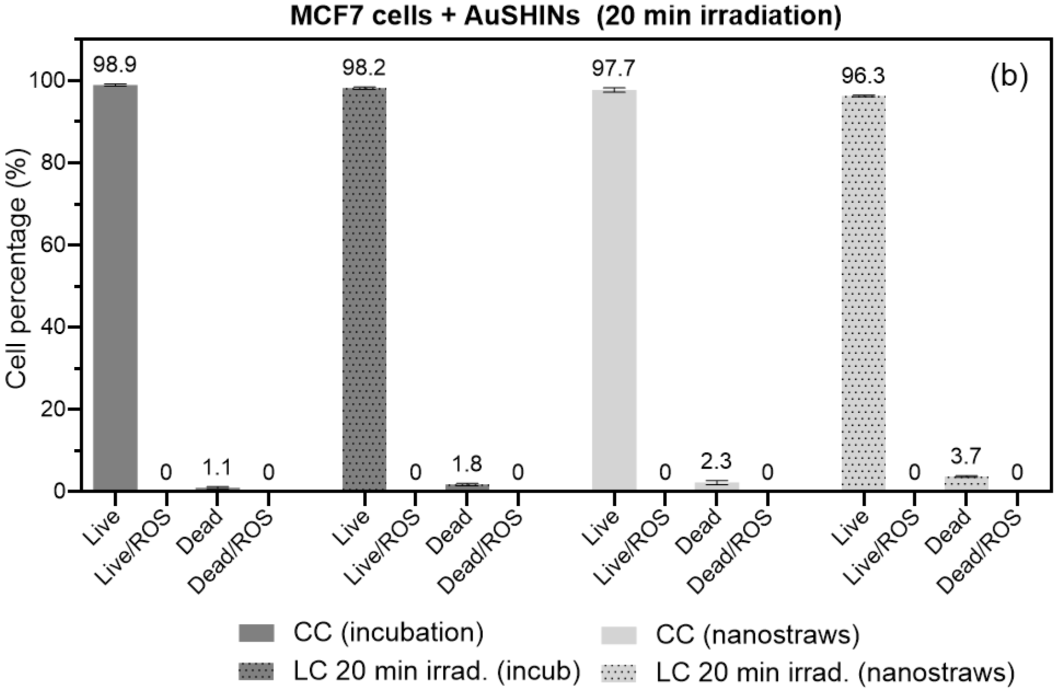

Figure S3. Percentage of cells that are alive without ROS (Live), alive with ROS (Live/ROS), dead without ROS (Dead), and dead with ROS (Dead/ROS) for cellular controls (CC) and light controls (LC) for (a) MDA-MB-231 cells and (b) MCF7 cells. Cellular controls are cells seeded in 96-well plates for 16 h not exposed to light irradiation (CC (incubation)) and cells seeded on nanostraw substrate, subjected to an injection of PBS devoid of AuSHINs and not exposed to light irradiation (CC (nanostraws)). LC 20 min irrad. (incub) corresponds to cells seeded on 96-well plates and let adhered for 16 h before exposing them to 20 min irradiation at 525 nm (Power density = (4.8±0.2) mW/cm^2). LC 20 min irrad. (nanostraws) correspond to cells seeded on the nanostraw substrate, subjected to an injection of only PBS, before exposing them to 20 min irradiation at 525 nm.

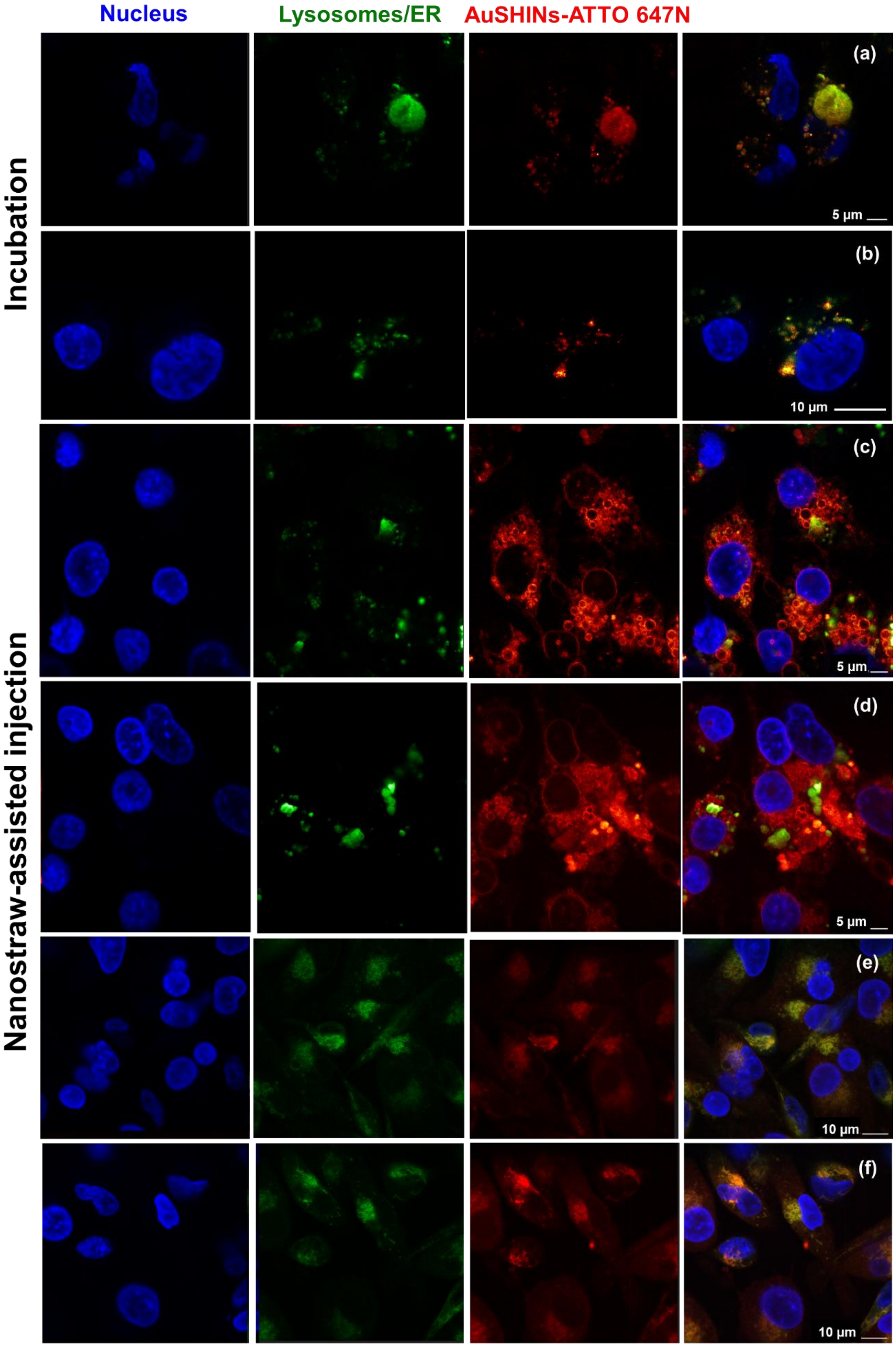

Figure S4. AuSHINs-ATTO 647N fate in MDA-MB-231 cells after (a) – (b) 2.0 h of incubation and (c) – (f) nanostraws-assisted injection imaged by confocal fluorescence microscopy. AuSHINs-ATTO 647N (in red), lysosomes (green in (a), (b), (c) and (d), stained with Lysotracker), ER (green in (e) and (f), stained with ER Tracker) and nucleus (blue, labelled with Hoechst 3342).

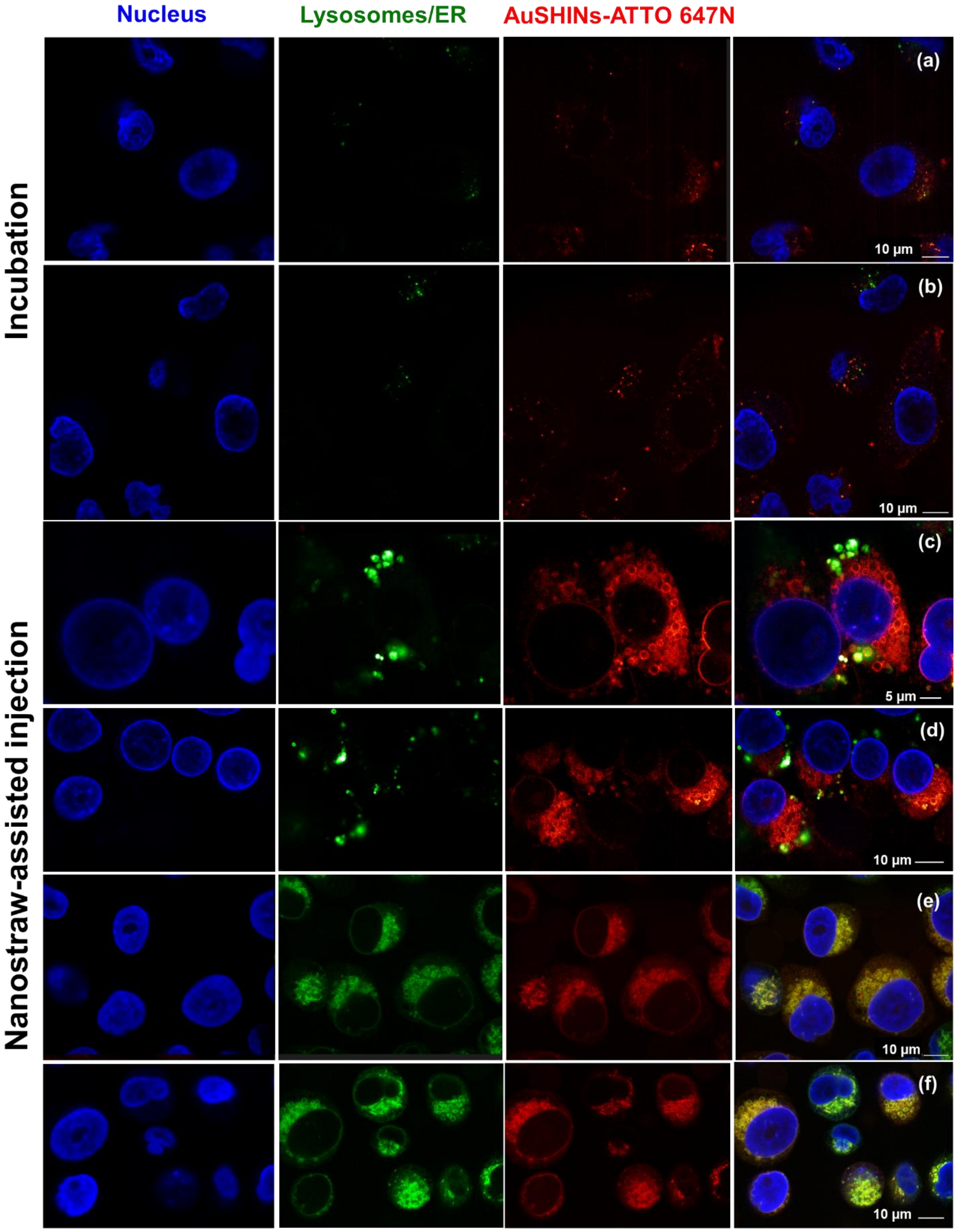

Figure S5. AuSHINs-ATTO 647N fate in MCF7 cells after (a) – (b) 2.0 h of incubation and (c) – (f) nanostraws-assisted injection imaged by confocal fluorescence microscopy. AuSHINs-ATTO 647N (in red), lysosomes (green in (a), (b), (c) and (d), stained with Lysotracker), ER (green in (e) and (f), stained with ERTracker) and nucleus (blue, labelled with Hoechst 3342).
